# ECDC launches public consultation on rotavirus vaccination in infancy

**DOI:** 10.2807/1560-7917.ES.2016.21.42.30374

**Published:** 2016-10-20

**Authors:** Eurosurveillance editorial team

**Affiliations:** 1 European Centre for Disease Prevention and Control (ECDC), Stockholm, Sweden

**Keywords:** Rotavirus, vaccination

On 17 October, the European Centre for Disease Prevention and Control (ECDC) launched a public consultation on the preliminary scientific advice document ‘Expert opinion on rotavirus vaccination in infancy’. The consultation is open until 28 November 2016.

The aim of the consultation is to harvest input for the ECDC expert opinion which should provide European Union/European Economic Area Member States with scientific opinion and expert opinion to support the decision-making process on the possible introduction and monitoring of routine vaccination against rotavirus-induced gastroenteritis in infants.

Please read more here on how to submit contributions.

